# Investigation on a Freeze-Drying Process for Long-Term Stability of mRNA-LNPs

**DOI:** 10.3390/vaccines14030242

**Published:** 2026-03-06

**Authors:** MD Faizul Hussain Khan, Ayyappasamy Sudalaiyadum Perumal, Amine A. Kamen

**Affiliations:** 1 Viral Vectors and Vaccines Bioprocessing Group, Department of Bioengineering, McGill University, Montreal, QC H2X 1Y4, Canada; md.f.khan@mail.mcgill.ca; 2 Department of Bioengineering, McGill University, Montreal, QC H3A 0E9, Canada; ayyappasamy.sudalaiyadumperumal@mcgill.ca

**Keywords:** freeze-drying, lyophilization, mRNA vaccines, lipid nanoparticles (LNPs), mRNA-LNPs, formulation optimization, cryoprotectants, stabilizers, buffers, physicochemical stability, long-term storage, cold chain, refrigerated storage, vaccine delivery

## Abstract

**Background**: Thermostability remains a key bottleneck for equitable access to mRNA-LNPs vaccines, mainly due to cold-chain requirements. **Objectives and methods**: Here, we optimized freeze-drying formulations by screening excipients (sugars, sugar alcohols, and proteins) and buffers to preserve mRNA-LNPs as solid formulations under ambient and refrigerated conditions. Physicochemical properties (size, polydispersity index [PDI], and encapsulation efficiency [EE]) and functional integrity, assessed by fluorescence-based *in vitro* transfection assays, were evaluated during long-term storage of up to six months. **Results**: Preliminary screening identified 20% sucrose and trehalose with Tris or histidine buffers as optimal for preserving physicochemical properties during freeze-drying, including high encapsulation efficiency (>90%), particle size (~200 nm), and low polydispersity (PDI < 0.2). Mannitol, gelatin, and PBS-based buffers showed adverse effects. At 4 °C, formulations F1–F3 maintained physicochemical stability and functional transfection activity for up to four months. In contrast, 20 °C storage caused progressive destabilization, with increased size, PDI, and encapsulation loss (>60% by six months). Among all formulations, 20% sucrose with 5 mM Tris (F1) showed the most robust preservation of physicochemical integrity and *in vitro* transfection efficiency under refrigerated and ambient conditions. **Conclusions**: Sugars outperformed sugar alcohols and gelatin as cryoprotectants. All formulations were stable, including functionally active at 4 °C for up to four months, while a sucrose/Tris formulation retained acceptable stability at 20 °C. Overall, the results demonstrate the feasibility of storing mRNA drug products as solid formulations at non-freezing temperatures.

## 1. Introduction

After the success of COVID-19 vaccination campaigns, mRNA-LNPs vaccines have emerged as a next-generation platform for disease prevention and immunization. This technology offers new opportunities to address previously untreatable conditions, emerging pathogens, and orphan or rare diseases, and also enables therapeutic applications such as the delivery of CRISPR–Cas9 gene-editing systems [1,2]. Unlike other vaccine types that require complex and expensive manufacturing facilities to meet strict safety standards, the mRNA vaccine platform is more techno-economically feasible and can be rapidly scaled for large-scale production [3,4,5,6]. However, one of the critical bottlenecks is the poor stability of the core mRNA molecule (therapeutic component) and the lipid nanoparticles (LNPs) delivery system [7,8]. This limited thermostability of the mRNA vaccines and their need for ultra-cold storage significantly hinders large-scale distribution and global delivery, particularly in low-resource regions [7,8].

The genetic information encoded within mRNA is inherently prone to degradation through hydrolysis and oxidation [9]. The dominant pathway of mRNA degradation is the hydrolysis in the presence of water. This ultimately affects the base sequence and secondary structure of the mRNA [10]. On the other hand, oxidative damage breaks the mRNA strands, compromising the vaccine efficacy [11]. Also, the presence of nucleases can degrade the mRNA molecule and make it functionally inactive. Vaccine stability is also dependent on the molecular integrity of the mRNA sequence, including the 5′ cap, untranslated regions (UTRs), coding regions, and the poly-A tail, which are critical for successful translation [8,10]. Modifications in these regions can greatly affect the stability of mRNA.

Further, LNPs that encapsulate mRNA serve a two-fold purpose: to aid as a delivery vehicle for the mRNA cargo into target cells and facilitate endosomal escape while also acting as a physical barrier for nucleases and other degradative factors outside the cell [2,12]. But the LNPs are also prone to stability issues, due to aggregation, fusion, or collapse, leading to an increase in particle size or polydispersity index (PDI) of the LNPs [13]. Several factors, including exposure to light, oxygen, metal ion residues, lipid composition, structure, pH, temperature, and buffer, influence this instability [14].

Proper storage conditions are required to maintain the stability of mRNA vaccines. For example, Pfizer’s COVID-19 vaccine needs to be stored at −70 °C and Moderna’s COVID-19 vaccine at −20 °C [3]. To improve the thermostability of mRNA-LNP vaccine, current research focuses on LNP formulations and on advances in formulation strategies [15]. In this context, freeze-drying of mRNA vaccines has the potential to maintain stability at relatively higher temperatures. This is considered an effective process for mRNA vaccine formulation, converting the unstable liquid form into a stable dry powder. This might bypass the strict requirement for storage and transport at ultra-low temperatures [16].

The freeze-drying process comprises several controlled steps. Initially, the mRNA-LNPs are mixed with appropriate excipients (such as cryoprotectants and buffers), and freezing is done at a very low temperature. In the second step, primary drying is performed by applying a vacuum to sublimate water from the mRNA-LNPs formulation. A subsequent secondary drying step gently removes residual water by raising the temperature (up to around 20–40 °C) [1,3,17].

While converting the liquid formulation into a solid formulation by freeze-drying to remove water content seems a plausible approach, the freeze-drying process itself introduces additional in-process stresses that can degrade or destabilize the LNPs and the mRNA therapeutic cargo [18]. The choice of formulation components is vital for a successful freeze-drying cycle. Optimally chosen excipients play a major role in preserving the integrity and prolonging the shelf life of mRNA-LNPs during freeze-drying and after freeze-drying. Certain excipients, mainly sugars and buffers, are used to counteract the process-associated stress, such as excessive moisture [19]. Moisture, if not optimally controlled, might lead to the hydrolysis of the mRNA cargo, as observed by several studies [20]. Sugars such as sucrose and maltose play a protective role by stabilizing the LNP structure during freeze-drying and reconstitution [16,21]. Tris buffer is usually preferred over PBS because the latter induces mRNA leakage during drying [3,16,19]. Optimizing lipid ratios in LNPs is also critical for maintaining the stability of mRNA-LNPs vaccine [1,22].

In this study, we evaluated the effect of different excipients and buffers on the freeze-drying process of mRNA-LNPs. This evaluation guided the selection of optimized formulations for long-term stability at ambient conditions and at 4 °C. These optimized formulations were tested for physicochemical assays to evaluate mRNA-LNP stability and *in vitro* cell-based (HEK-293T) transfection assay to assess transfection efficiency for up to 6 months under different storage conditions. The results of this study demonstrate the development of an efficient freeze-drying process to achieve long-term stability of the mRNA drug product. The findings will ultimately contribute to extending the shelf life of mRNA-LNPs in less stringent cold-chain conditions.

## 2. Materials and Methods

### 2.1. Preparation of mRNA-LNPs

Capped mCherry-mRNA transcripts were purchased from TriLink Technologies (San Diego, CA, USA). The ionizable lipid ALC-0315 and PEG-lipid ALC-0159 were obtained from Cayman Chemical (Ann Arbor, MI, USA), whereas cholesterol and DSPC were sourced from Millipore Sigma (Burlington, MA, USA). Lipids were resuspended in 100% ethanol at a defined molar ratio of 50:10:38.5:1.5, corresponding to ALC-0315, 1,2-distearoyl-sn-glycero-3-phosphocholine (DSPC), cholesterol, and ALC-0159, respectively.

In parallel, the mRNA (25 µg/mL) was diluted in sodium acetate buffer with a pH of 5.0. The lipid-to-mRNA weight ratio was carefully maintained at 10:1 to ensure optimal encapsulation efficiency. mRNA encapsulation into lipid nanoparticles (LNPs) was achieved by manual syringe mixing, where the ethanol-based lipid phase was mixed with the aqueous phase of the mRNA solution. Following encapsulation, the resulting mRNA-LNPs solution was subjected to dialysis using Slide-A-Lyzer™ Dialysis Cassettes (10 kDa MWCO; Thermo Fisher Scientific, Waltham, MA, USA). Dialysis was carried out using 200× volume of 5 mM Tris buffer (pH 8.0) at 4 °C for 2 h to exchange ethanol with the buffer.

### 2.2. Freeze-Drying

Preliminary formulations were prepared in the early stages of this study, as outlined in Table 1. The excipients used included sucrose, trehalose, and mannitol (ranging from 1% to 20%), gelatin (0.5% to 1%), Tris (5 mM), histidine (10 mM), and PBS (1X) at different concentrations. The optimized formulations identified in the preliminary study were used for the long-term study, as outlined in Table 2. The formulated mRNA-LNPs were prepared, filled into glass vials, and loaded into the Virtis Advantage Pro freeze dryer (SP Scientific, Warminster, PA, USA).

The encapsulated mRNA-LNPs were mixed with appropriate formulations (excipients and buffer) at a ratio of 1:1, where the final concentration of mRNA was 12.5 µg/mL. Each aliquot (200 µL) was filled into 1 mL sterilized glass vials (WHEATON^®^, Millville, NJ, USA) and sealed with butyl stoppers. The cycle included freezing at −50 °C for 5 h, primary drying at −30 °C for 5 h, and secondary drying for 1 h with gradual temperature increase (Table 3). The lyophilization cycle parameters were selected based on the glass transition temperature of the respective formulations, as well as prior studies [18,21] that identified optimized conditions for mRNA-LNPs. Vials were stoppered under vacuum without inert gas backfilling.

### 2.3. Stability Study

All the freeze–dried formulations containing mRNA-LNPs were kept at different time points (0 to 6 months) at various storage conditions (−80, −20, 4, and 20 °C). After specific time points, the freeze–dried cakes were resuspended in RNA-free water, and the size, PDI, EE, and transfection efficiency were assessed. After reconstitutions, these formulations were characterized for material properties based on size distribution, polydispersity index, and encapsulation efficiency.

### 2.4. mRNA-LNPs Characterization

#### 2.4.1. Encapsulation Efficiency (EE%)

The encapsulation efficiency of mRNA was quantified using the protocol of the Quant-iT RiboGreen RNA Assay Kit (Thermo Fisher Scientific, Waltham, MA, USA). The mRNA-LNPs samples were diluted in 1 x TE buffer to measure unencapsulated RNA or in 2% Triton X-100 to measure the total RNA content. To disrupt the LNPs, the plate was incubated with 2% Triton X-100 for 10 min at 37 °C, then RiboGreen dye was added. A standard curve was established using the ribosomal RNA standards supplied with the kit. Fluorescence intensity was then recorded using an Agilent BioTek Synergy HTX MultiMode Microplate Reader (Agilent Technologies, Santa Clara, CA, USA).

#### 2.4.2. Particle Size and Polydispersity Index (PDI)

The particle size and PDI of the mRNA-LNPs were determined by dynamic light scattering (DLS) on a Zetasizer Ultra (Red Label) (Malvern Panalytical, Malvern, UK). Samples were equilibrated to room temperature, with a refractive index of 1.37, and measured in 50 µL disposable cuvettes (Sarstedt Inc., Nümbrecht, Germany) to determine size and PDI.

#### 2.4.3. Capillary Electrophoresis for mRNA Integrity

After freeze-drying, mRNA-LNPs samples were incubated with 0.1% Triton X-100 at 37 °C for 10 min to disrupt the LNPs and release encapsulated mRNA. The resulting samples were subsequently diluted tenfold. Digital electrophoresis was performed using an Agilent 2100 Bioanalyzer with the RNA 6000 Pico Kit (Agilent Technologies, Santa Clara, CA, USA) following the manufacturer’s protocol. mRNA integrity was assessed by calculating the proportion of the main peak, expressed as the percentage of the total electropherogram area corresponding to the ~1000 nt peak.

### 2.5. In Vitro Assay for mRNA-LNPs

HEK293 cells were seeded into 96-well plates and cultured for 24 h to allow cell attachment and growth. Following this, the cells were transfected with freeze–dried mRNA-LNP formulations and the control (without formulations). The transfected cells were then incubated for an additional 24 h at 37 °C with 5% CO_2_ to enable mRNA uptake and expression. After the incubation period, all the wells were imaged using an inverted Olympus IX83 confocal microscope to assess fluorescence intensity, indicating successful transfection of the mRNA-LNPs. The images from the microscope were processed and analyzed as described elsewhere [23] using ImageJ FIJI (version 1.54f) [24] to estimate the time point at which the transfection expression was not observed, indicating the lack of functional mRNA in the LNP-excipient sample. Transfection efficiency was quantified from fluorescence microscopy images using an ImageJ-based analysis pipeline. The ratio of fluorescent cells to the total cell population was calculated to determine microscopy-derived transfection efficiency. Cell segmentation and counting were performed using ImageJ’s built-in thresholding and particle analysis functions.

### 2.6. Statistical Analysis

Statistical analyses were conducted using one-way analysis of variance (ANOVA) in GraphPad Prism (version 9.4.1). Comparisons between the freshly prepared mRNA-LNPs stock and the freeze–dried formulations were performed using Dunnett’s post hoc test with a 95% confidence interval. Differences were considered statistically significant at *p* < 0.05 and are denoted by asterisks (*).

## 3. Results

### 3.1. Preliminary Screening of Excipients During the Freeze-Drying of mRNA-LNPs

**Figure 1:**
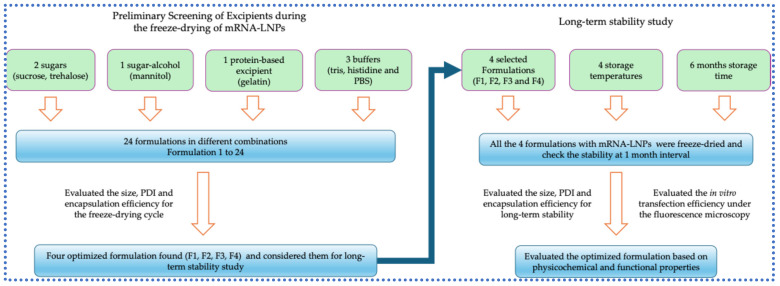
Schematic describing the experimental design and process workflow.

A range of excipients, namely two sugars—sucrose and trehalose, a sugar-alcohol—mannitol, and a protein-based lyoprotectant—gelatin, were combined with three different buffers—Tris, histidine, and PBS, in different combinations, and were assessed for freeze-drying mRNA-LNPs (Table 1). All these formulations were freeze–dried, reconstituted immediately, and the physical parameters, including size, PDI, and encapsulation efficiency (Figure 1), were evaluated. The goal at this point was physicochemical evaluation of the best formulation for subsequent long-term storage studies above zero, ambient temperatures (4 and 20 °C).

The experimental plan is shown in Figure 1. Further, though we tested several percentages of the cryoprotectants (shown in Table 1) for different formulations, we did not test beyond 20% of sugar or sugar alcohols, as high cryoprotectant levels will lead to excessive viscosity of the injectable formulated product, leading to side reactions like pain during administration. Also, it could contribute to crystallization and other undesirable effects during the optimization of the freeze-drying process. Further, the higher the percentage of the sugars or sugar alcohols, the better the physicochemical stability was achieved at the end of the free-drying cycles.

**Figure 2:**
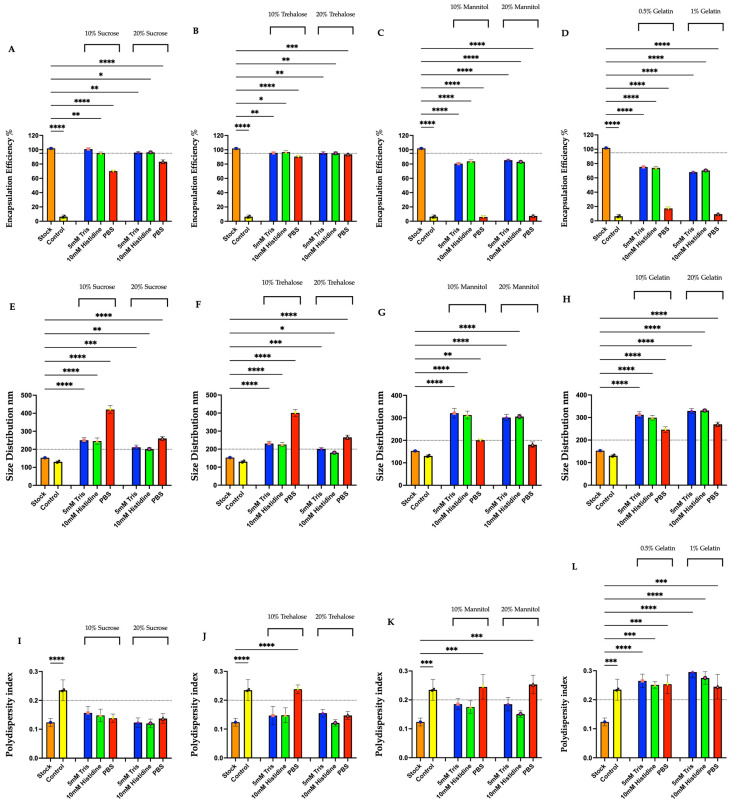
Multiple freeze–dried mRNA–LNP formulations incorporating distinct cryoprotective sugars and buffering systems were evaluated for encapsulation efficiency, hydrodynamic diameter, and polydispersity index (PDI). Statistical comparisons between freshly prepared (stock) and freeze–dried formulations were conducted using one-way analysis of variance (ANOVA). Statistical significance is indicated by asterisks, with thresholds defined as *p*-values less than 0.05 (**** *p* < 0.0001; *** *p* < 0.001; ** *p* < 0.01, * *p* < 0.05).

All the formulations, including the control was compared to that of the stock mRNA-LNPs with respect to encapsulation efficiency (%), LNP size (nm), and PDI measurements. The result shown in Figure 2 demonstrates that the LNP preparations in control destabilized and collapsed to near zero encapsulation efficiency without cryoprotectant, a significant reduction in encapsulation efficiency (****, *p* < 0.0001). Among the three buffers tested at different molarity concentrations, the 5 mM Tris and 10 mM histidine buffer showed high encapsulation efficiency compared to that of the PBS buffer. Tris and histidine exhibited high encapsulation efficiency (up to 95%) with both the excipients, sucrose and trehalose, at 10% and 20% concentration (Figure 2A,B). In contrast, PBS buffer showed very low efficiency with sucrose, but a comparatively better efficiency was found with trehalose, up to 95% (Figure 2A,B). Sugar-alcohol like mannitol showed around 20% loss in both the Tris and histidine buffer. Another combination of mannitol with PBS showed a 90% loss in encapsulation efficiency (Figure 2C). Also, the same trend was observed with gelatin in Tris and histidine buffer, with a 30% loss in encapsulation efficiency. Like mannitol, gelatin also showed a significant drop in encapsulation efficiency with PBS buffer, with a loss of around 80% to 90% (Figure 2D).

Also, the size distribution of lipid nanoparticles varied noticeably with buffer and sugar concentration. In 10% sucrose, Tris, and histidine produced particles around 240 nm, while PBS yielded much larger particles around 400 nm (Figure 2). At 20% sucrose, both Tris and histidine yielded smaller particles around 200 nm, whereas PBS resulted in relatively large particles around 260 nm (Figure 2E). Also, trehalose formulations displayed a similar trend in particle size distributions. At 10% trehalose, Tris and histidine generated particles roughly around 220 nm, while PBS produced the largest size at about 400 nm. Increasing trehalose concentration to 20% with Tris and histidine maintained particle sizes (~200 nm and ~180 nm, respectively), but PBS yielded larger particle sizes (~300 nm) (Figure 2F). Again, the buffer played a major role with mannitol, where 10 and 20% mannitol with Tris and histidine resulted in particle sizes around 300 nm, while PBS, unlike, yielded particles near 170–200 nm (Figure 2G). The gelatin with buffer also showed an adverse impact on size distribution. With 0.5 and 1% gelatin, Tris and histidine-maintained particles of ~300 nm, and PBS yielded sizes near 250–280 nm (Figure 2H).

From the PDI data, the results demonstrate that the choice of stabilizer and buffer also affects the size homogeneity (i.e., uniformity of particle size) of the freeze–dried mRNA-LNPs samples. Samples with sucrose, trehalose, mannitol, and gelatin showed lower PDI values (<0.2), especially when formulated with Tris and histidine buffers, indicating good homogeneity and stability (Figure 2I–L). In contrast, mannitol and gelatin formulations, particularly those containing PBS buffer, exhibited higher PDI values (>0.2) (Figure 2K,L). Overall, all these excipients combined with Tris and histidine buffer maintained a more consistent particle distribution.

Overall, the data demonstrated that both buffer type and excipient concentration play a vital role in maintaining the encapsulation efficiency. Among the buffers, Tris and histidine looked more promising than PBS. Among the sugars, sugar-alcohols, and protein-based cryoprotectants tested, sugar (sucrose and trehalose) showed better results in terms of encapsulation efficiency, size, and PDI compared to the sugar alcohols and proteins. From the above series of physicochemical analyses, the four best formulations were tested further for the next stages of long-term storage studies.

**Figure 3:**
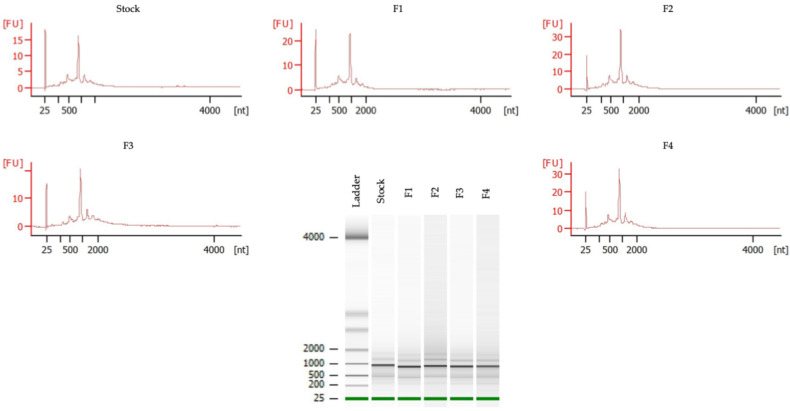
Capillary electrophoresis for RNA integrity analysis of the stock of mRNA-LNPs and four freeze–dried mRNA-LNPs formulations, including F1, F2, F3, and F4. The percentage of the area of the main peak (1000 nt) relative to the total RNA detected.

The four selected formulations, F1 (20% sucrose + 5 mM Tris), F2 (20% sucrose + 5 mM histidine), F3 (20% trehalose + 5 mM Tris), and F4 (20% trehalose + 5 mM histidine), found from the preliminary screening, were also evaluated to check the mRNA integrity (Figure 3). The Bioanalyzer analysis shows that mRNA integrity is well preserved in all freeze–dried mRNA-LNP formulations (F1–F4). This is evidenced by the presence of a dominant full-length mRNA peak (1000 nt) comparable to that of the freshly prepared stock mRNA-LNPs. Particularly, no high-molecular-weight aggregates or abnormal species were detected. This result indicates that optimized freeze–dried formulations did not allow RNA aggregation, concatemer formation, or irreversible LNP fusion. The consistency of electropherogram profiles among F1–F4 further supports good formulation robustness and reproducibility.

### 3.2. Long-Term Stability Study

From the preliminary study shown above, the four formulations labeled as F1, F2, F3, and F4 were selected for a long-term stability study (Figure 1, Table 2). Also, one liquid control, which was not lyophilized, was kept during the stability study at every temperature and every storage time point as a control. The long-term stability results present the stability characterization using particle size, polydispersity index (PDI), and encapsulation efficiency of mRNA-loaded lipid nanoparticles (mRNA-LNPs) stored under different temperatures (−80 °C, −20 °C, 4 °C, and 20 °C).

**Figure 4:**
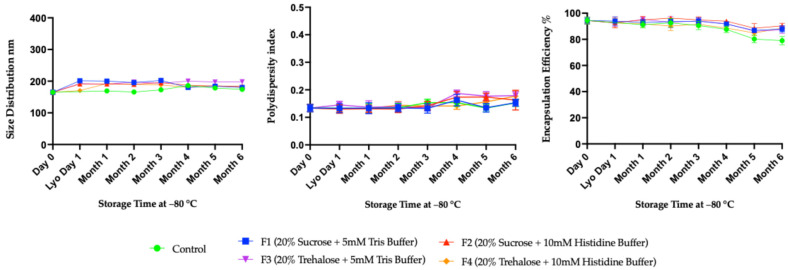
Long-term stability data using four formulations of mRNA-LNPs at −80 °C. These formulations were characterized at one-month intervals using DLS and the Ribogreen assay to assess size distribution, polydispersity index (PDI), and encapsulation efficiency. All assays were done in biological replicates of *n* = 3.

The freeze–dried mRNA-LNPs formulations stored at −80 °C demonstrated good physical stability, as indicated by particle size measurements across all samples. The mean size distribution remained consistently approximately 200 nm over six months of storage (Figure 4, left). This indicates that there was no major aggregation or fusion of nanoparticles over the stability period. Polydispersity index (PDI) analysis further supports the robustness of these formulations. Throughout the six-month storage period, the PDI values for all samples remained low, generally between 0.12 and 0.20, indicating that there was no major deviation in the distribution of the particle size throughout storage (Figure 4, middle). These findings indicate that lyophilization and storage at −80 °C did not produce significant heterogeneity within the lipid nanoparticle population. Also, the encapsulation efficiency data confirm that mRNA cargo retention within lipid nanoparticles was maintained during long-term storage (Figure 4, right). The encapsulation efficiency remained high in all freeze–dried formulations stored at −80 °C, consistently ≥90% over six months. However, a decline of about 5% was observed towards the end of the 6-month time points in these freeze–dried formulations. This loss was limited and did not compromise the overall encapsulation performance, compared with the 10–15% loss observed in the liquid control. All these data collectively demonstrate that freeze–dried mRNA-LNPs maintained the key physicochemical properties at −80 °C throughout the study period of more than 6 months.

**Figure 5:**
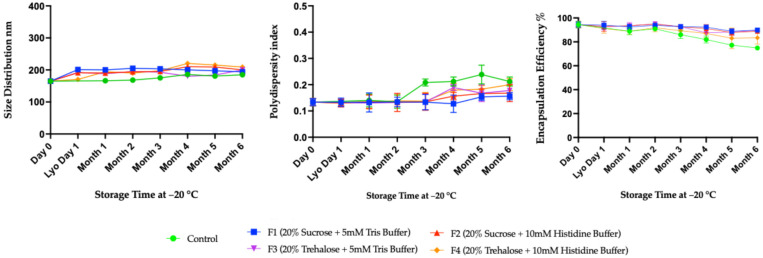
Long-term stability data using four formulations of mRNA-LNPs at −20 °C. These formulations were characterized at one-month intervals by DLS and Ribogreen assay to observe the size distribution, PDI, and encapsulation efficiency. All assays were done in biological replicates of *n* = 3.

The stability assessment of mRNA-LNPs stored at −20 °C demonstrated that all formulations maintained relatively stable particle size (Figure 5, left) and PDI (Figure 5, middle) over time, although the control showed increases in both size and PDI, due to the lack of excipients. Particle size distributions remained predominantly around 200 nm across all formulations, indicating that the freeze–dried formulations prevented aggregation or fusion of LNPs. Polydispersity index (PDI) analysis also reveals that most formulations maintained robust homogeneity, with PDI values below 0.20 throughout the storage period (Figure 5, middle). In contrast, the control showed an increase in PDI after four months, peaking at 0.25 at six months, indicating substantial heterogeneity and potential particle destabilization. Further, the encapsulation efficiency of control at −20 °C declined more drastically than in ultra-low storage, dropping below 80% after six months (Figure 5, right). The decline in EE% began after 2 months at −20 °C. Formulations containing sucrose or trehalose retained encapsulation efficiency (Figure 5, right) around 90% for up to six months, except the F4 formulation, which had 10 mM histidine instead of 5 mM Tris. Overall, these findings confirm that lyophilized mRNA-LNP formulations F1, F2, and F3 were stable at −20 °C during the entire 6 months of observation (Figure 5).

**Figure 6:**
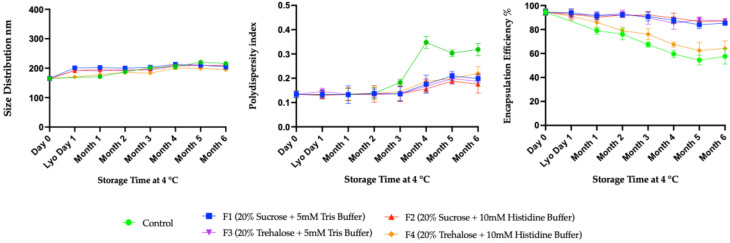
Long-term stability data using four formulations of mRNA-LNPs at 4 °C. These formulations were characterized at one-month intervals by DLS and Ribogreen assay to observe the size distribution, PDI, and encapsulation efficiency. All assays were done in biological replicates of *n* = 3.

Figure 6 shows mRNA-LNP particles and their physicochemical properties at 4 °C. Here, all formulations initially exhibited similar particle sizes up to 3 months. However, as storage time progressed, the control showed a more pronounced increase in particle size (~230 nm) than the other formulation (Figure 6, left). Except for F4, the other three freeze–dried formulations, F1, F2, and F3, maintained constant size distributions throughout the six months. In the PDI graph (Figure 6, middle), all formulations retained low PDI values, indicating a sustained, homogeneous nanoparticle population over time. In contrast, the control’s PDI sharply increased within four months (>0.3), indicating greater size heterogeneity likely due to aggregation or fusion (Figure 6, middle). The PDI data emphasized that freeze–dried formulations with sucrose or trehalose as cryoprotectants, in Tris and histidine buffers, effectively maintained both size stability and particle uniformity during long-term storage. Also, the encapsulation efficiency tended to decrease with storage time, although the three formulations, F1, F2, and F3, maintained their encapsulation efficiencies over the 6 months of the stability study (Figure 6, right). The most pronounced decline was observed in the control group, falling below 60% by six months in the absence of stabilizing excipients. The next highest loss was observed in the F4 formulation containing 20% trehalose and 10 mM histidine. This suggests that mRNA integrity and encapsulation decline over time in the absence of appropriate formulation, whereas inclusion of suitable cryoprotectants and excipients conclusively enhances stability. In summary, these results collectively illustrate that LNPs formulations containing sucrose or trehalose with Tris and sucrose with histidine buffer (F1, F2, and F3) were found most effective in maintaining size, dispersity, and encapsulation efficiency during long-term storage at 4 °C. These trends validate the critical role of optimized cryoprotectant and buffer combinations in enhancing the shelf life of mRNA-LNPs.

**Figure 7:**
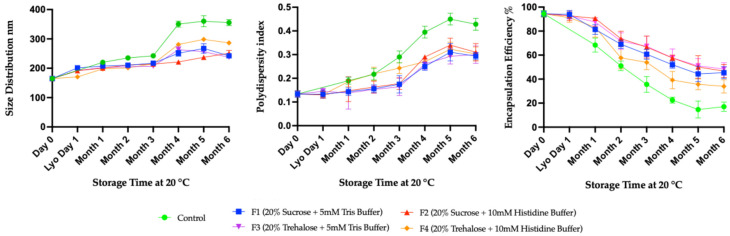
Long-term stability study using four formulations of mRNA-LNPs at 20 °C. These formulations were characterized at one-month intervals by DLS and Ribogreen assay to observe the size distribution, PDI, and encapsulation efficiency. All assays were done in biological replicates of *n* = 3.

The results at 20 °C demonstrate a different representation of the stability of freeze–dried mRNA-LNPs over six months (Figure 7). The size and PDI at 20 °C showed more marked increases compared to 4 °C, highlighting that higher temperatures accelerate mRNA-LNPs instability (Figure 7, left and middle). For size distribution, the control exhibited a sharp increase after three months, exceeding 350 nm. But all four formulations (F1–F4) maintained comparatively smaller size over six months. Here, the F1, F2, and F3 retain the particle sizes below 220 nm, while the other formulation, F4, exceeds particle sizes above 250 nm. The PDI also followed a similar trend. The control group showed a marked increase in polydispersity from the first month, reaching 0.4 at six months (Figure 7, middle). Such high polydispersity indicated that the particles became more heterogeneous, likely due to aggregation. In contrast, the four formulations kept the PDI below 0.2 for 3 months, indicating more uniformity and homogeneity. After 3 months, all the freeze–dried formulations showed a sharp increase, reaching a PDI of 0.3 over 6 months. This indicates that the freeze–dried formulations could not maintain the homogeneity at this elevated temperature for a long time, likely again due to aggregation or fusion of LNPs. At 20 °C, the encapsulation efficiency dropped rapidly in the control, falling below 20% at six months (Figure 7, right). This indicates poor retention of the mRNA payload within the LNPs. Also, major loss was observed in all the freeze–dried formulations, retaining between 40% and 60% efficiency at six months. Overall, the data showed the clear instability of lipid nanoparticles at 20 °C and the ineffectiveness of freeze–dried formulations in maintaining physical integrity and encapsulation efficiency.

In addition to physicochemical studies, key evidence of mRNA integrity under storage conditions, in the presence of excipients, and at different temperatures is provided by *in vitro* transfection assays. The main goal of the *in vitro* transfection assays is to evaluate the integrity of mRNA encapsulated within LNPs across excipient combinations and to determine whether changes in the physicochemical properties of mRNA-LNPs excipient formulations stored at 4 and 20 °C affect transfection efficiency. Thus, the *in vitro* study assessing transfection efficiency used HEK293 cells and fluorescence microscopy. The red fluorescence signal corresponded to the expressed mRNA delivered by the LNPs, serving as a qualitative indicator of transfection within the cells.

**Figure 8:**
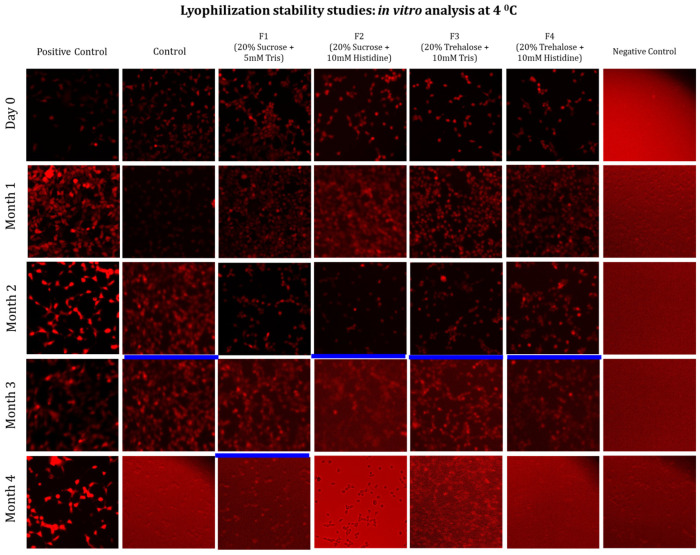
Long-term mRNA integrity test by *in vitro* transfection study using four formulations of mRNA-LNPs at 4 °C. These formulations were characterized at one-month intervals by measuring transfection efficiency using fluorescence microscopy. All assays were performed in biological replicates of *n* = 3, while the image presented above is a representative of one of the biological replicates. The blue line in each of the samples (across each column) represents the time point when transfection efficiency was lost, probably due to the mRNA-LNPs’ integrity being affected.

Figure 8 presents representative fluorescence microscopy images of cells transfected with the control (without any formulations) and freeze–dried formulations (F1–F4) up to 4 months of storage at 4 °C. Red fluorescence corresponds to reporter protein expression, reflecting the retained biological activity of each formulation, i.e., mRNA integrity. The stock formulation exhibited a high fluorescence signal for up to two months. Thereafter, the signal intensity progressively weakened, becoming undetectable by the fourth month (Figure 8). Notably, freeze–dried formulations F1, F3, and F4 showed consistently strong fluorescence signals for up to four months at 4 °C. This indicates that these formulations effectively maintained the biological functionality of the mRNA-LNPs during this time frame. In contrast, formulation F2 exhibited a weaker fluorescence signal at 4 months, suggesting major loss of possible structural integrity and reduced transfection efficiency after prolonged storage.

This finding highlights the superior performance of F1, F3, and F4, which preserved the functional activity up to four months, thereby demonstrating their suitability for long-term stabilization of the formulation.

**Figure 9:**
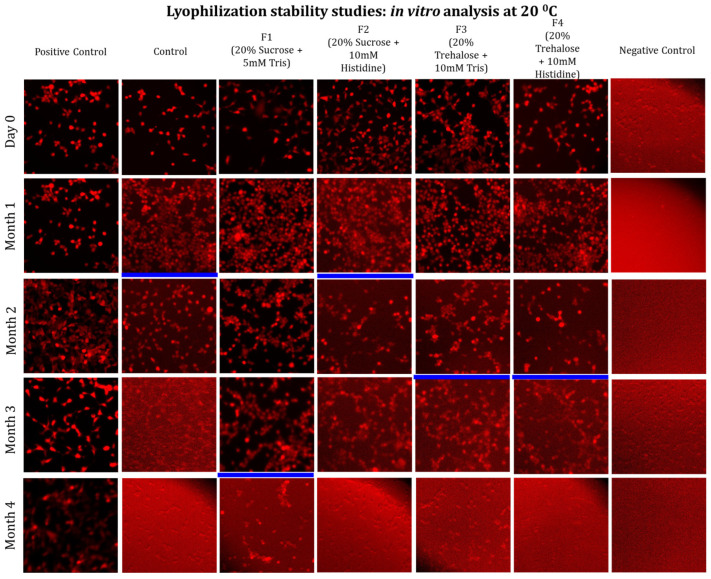
Long-term mRNA integrity test by *in vitro* transfection study using four formulations of mRNA-LNPs at 20 °C. These formulations were characterized at one-month intervals by measuring the transfection efficiency under fluorescence microscopy. All assays were performed in biological replicates of *n* = 3, while the image presented above is a representative of one of the biological replicates. The blue line in each of the samples (across each column) represents the time point when transfection efficiency was lost, probably due to the mRNA-LNP integrity being affected.

Again, fluorescence microscopy was performed for the freeze–dried formulations stored at 20 °C for 4 months (Figure 9). After 4 months of storage, formulation F1 exhibited a strong fluorescence with a reported intensity. In contrast, F2, F3, and F4 show reduced fluorescence intensity after 1 month. Also, the control started to show a very weak fluorescence signal after 1 month of storage, and the signal disappeared after 2 months of storage.

Supplementary Figure S1 presents the complete set of fluorescence microscopy images, including composite images for samples tested under different excipient conditions at two storage temperatures. Quantitative fluorescence image analysis (Supplementary Figure S2) confirmed the qualitative transfection trends observed in Figure 8 and Figure 9 and correlated well with physicochemical stability. Formulation F1 showed the highest and most sustained *in vitro* transfection efficiency, remaining >90% for up to four months at 4 °C before declining to ~50%. In contrast, storage at 20 °C resulted in a marked reduction in F1 transfection efficiency, falling below 30%. Other formulations exhibited lower and less stable transfection efficiencies, with F3 showing the next-best performance at 4 °C, maintaining activity for up to three months (~35%). Overall, these results demonstrate that formulation-dependent physicochemical stability directly translates to preserved biological functionality of mRNA-LNPs during storage.

**Figure 10:**
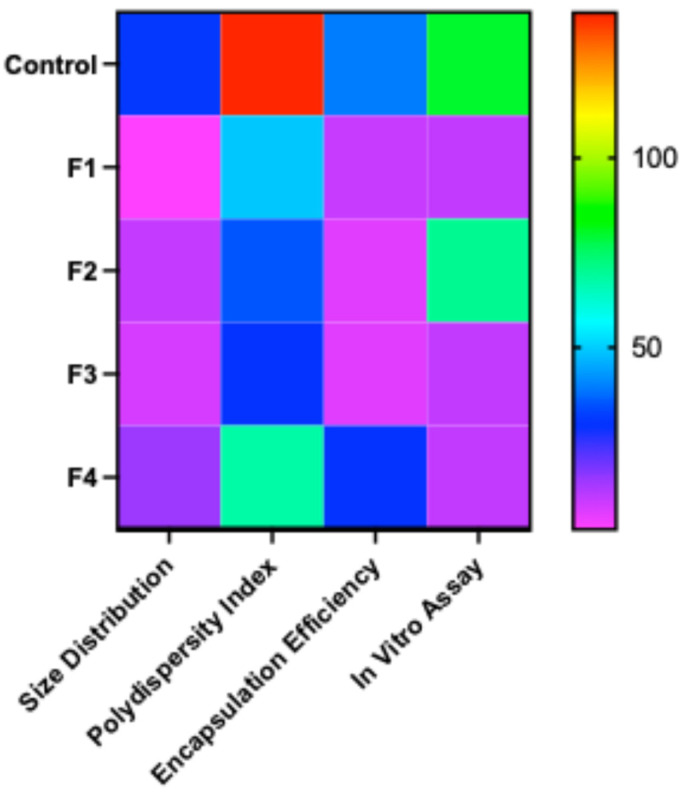
Heatmap illustrating the normalized comparative responses of different formulations at 4 °C across multiple physicochemical and functional parameters at the end of the stability studies, both physicochemical and *in vitro* assays. Each matrix element corresponds to a formulation–parameter pair, with the color gradient representing the relative percentage changes in the measured response. The heatmap uses a normalized color gradient in which pink/purple represents the lowest value changes in percentages, followed by blue–cyan (low to moderate), green (intermediate), and yellow–red (highest values).

To summarize the overall results of the storage stability studies, a heatmap demonstrated that freeze–dried mRNA-LNPs formulated with physicochemical attributes and biological activity during long-term refrigerated storage at 4 °C (Figure 10). The heatmap uses a normalized color gradient in which pink/purple represents the lowest value changes in percentages, followed by blue–cyan (low to moderate), green (intermediate), and yellow–red (highest values), enabling direct visual comparison among formulations. For size distribution and polydispersity index (PDI), pink and blue regions correspond to smaller and more homogeneous particles, whereas green to red regions indicate larger and more polydisperse systems. Here, F1, F2, and F3 indicate improved physical uniformity. Encapsulation efficiency increases from pink to blue, with the control and F4 showing relatively higher loss in encapsulation than F1, F2, and F3. In the *in vitro* study, the control and F2 display warmer colors, indicating substantial loss despite F2’s favorable size and PDI, while F1, F3, and F4 show retention of a good amount of fluorescence signal. Overall, the color gradient reveals that reduced particle size and PDI alone do not guarantee improved *in vitro* stability. This explains a disconnect between physicochemical properties and functional performance, underscoring the need to optimize formulations based on both parameters simultaneously.

## 4. Discussion

### 4.1. Impact of Manual Mixing on Initial mRNA-LNPs Physicochemical Properties

This study used manual mixing for mRNA-LNPs encapsulation, which, in general, provides slightly larger mRNA-LNPs particles, which corroborates well with our reported data here [25]. Also, the addition of excipients to the mRNA-LNPs will increase the effective size of the mRNA-LNPs, as previously reported elsewhere [26]. The observation here suggested that these were well reproduced in this study as well. While it is desirable to use microfluidic or Tee-junction-based encapsulation approaches, the current study employs a manual mixing approach for its simplicity and low-volume sample handling.

### 4.2. Freeze-Drying–Induced Stresses and Their Effects on mRNA-LNPs Stability

Freeze-drying subjected lipid nanoparticles to a combination of severe stresses, including ice crystal formation during freezing, dehydration during primary and secondary drying, and physicochemical perturbations during reconstitution. These stresses can induce changes in pH, ionic strength, and molecular organization, ultimately destabilizing LNPs structures. In the absence of appropriate protective excipients, such stresses frequently lead to particle aggregation, fusion, and leakage of encapsulated mRNA. The observed increases in particle size and polydispersity index (PDI) across several formulations following lyophilization underscore the sensitivity of mRNA-LNPs systems to freeze-drying conditions. These findings are consistent with prior studies reporting enlargement and heterogeneity of LNPs as a consequence of structural stress during dehydration [3,16,18,21].

### 4.3. Role of Excipients in Preserving Particle Size and Polydispersity During Lyophilization

Freeze-drying imposes severe freezing and dehydration stress on lipid nanoparticles. The preliminary screening of mRNA-LNPs formulations highlighted the significant impact of excipients and buffer on the physicochemical properties of lipid nanoparticles during the freeze-drying process. Formulations containing mannitol, gelatin, with Tris, histidine, and PBS buffer demonstrated higher particle size (up to 300 nm) and polydispersity index (PDI, >0.2) after freeze-drying (i.e., non-stable formulations). During freeze-drying, mRNA-LNPs are exposed to freeze-drying stresses such as freezing, dehydration, and reconstitution, which bring changes in pH or ionic strength and trigger partial fusion or aggregation of lipid nanoparticles. Ultimately, these stresses disrupt the stability of the LNPs by causing them to cluster together and resulting in an increased particle size after reconstitution. This is consistent with previous findings, where freeze–dried LNP formulations showed size enlargement due to structural stress during drying [3,16,18,21]. However, particle size and polydispersity index (PDI) were consistently monitored using dynamic light scattering (DLS) in this study, but incorporating additional characterization techniques, such as the transmission electron microscopy (TEM), to validate nanoparticle morphology and structural integrity, along with zeta potential analysis to better understand surface charge–driven colloidal stability and aggregation behavior during long-term storage, would further strengthen the physicochemical assessment of these formulations. Therefore, these complementary analyses will be prioritized in our future work to ensure higher reliability and a more comprehensive evaluation of LNP stability.

### 4.4. Structural Rearrangements of Lipid Nanoparticles During Freeze-Drying

Also, during freezing, ice crystallization and water removal cause phase separation and mechanical disruption of the lipid bilayer. This ultimately forces lipids to reorganize and form a bleb-like structure on the nanoparticle surface [17,19,21]. Lyophilization-induced blebs are typically depleted of encapsulated mRNA because they form after particle assembly and loading, which has been associated with reduced transfection efficiency [19,27]. These blebs typically exclude encapsulated mRNA, as their formation occurs after initial assembly and loading steps. In contrast, blebs formed during LNP self-assembly retain mRNA and preserve functional activity [28]. In the present study, control formulations without excipients showed minimal changes in particle size after freeze-drying but exhibited a marked reduction in encapsulation efficiency (<10%), a trend consistent with reported lyophilization-induced bleb formation in the literature. While bleb formation was not directly visualized here, these observations support a mechanistic interpretation based on prior studies. Importantly, formulations containing sucrose or trehalose with Tris or histidine buffers preserved encapsulation efficiency, size, and PDI during freeze-drying, suggesting effective mitigation of structural perturbations associated with mRNA loss.

### 4.5. Mechanistic Basis of Encapsulation Efficiency Loss in Excipient-Free Formulations

Also, there was a marked reduction in encapsulation efficiency found in the control without excipients, paralleling results by Muramatsu et al. (2022), who reported that excipient-free liquid formulation suffers severe encapsulation loss in the freeze-drying cycle as well as during storage [29]. When the process is carried out without excipients, the nanoparticles are exposed to mechanical and chemical stresses. The absence of excipients magnifies the risk of irreversible damage (due to the stripping of essential water molecules and mechanical collapse). These stresses disrupt lipid nanoparticles, which ultimately leads to the leakage of the encapsulated mRNA [2,3,16,30,31]. In contrast, optimized freeze–dried formulations provide a stabilizing matrix that helps to preserve nanoparticle shape, size, and the integrity of their mRNA payload through each stage of drying and subsequent storage [3,16,32,33,34].

### 4.6. Sugars Are Better Cryoprotectants for mRNA-LNPs than Sugar Alcohols and Proteins (Gelatin)

This study showed that sucrose and trehalose effectively preserved encapsulation efficiency and particle size distribution throughout freeze-drying. This effect might be attributed to their ability to increase the lipid denaturation temperature as well as the glass transition temperature [3,18,21]. Sucrose binds more directly to the surface of the LNPs to maintain stability at higher temperatures and lower water content [35]. However, trehalose shows more thermal stability under low-temperature freeze-drying conditions. In contrast, mannitol can lead to an increased particle size and lower encapsulation efficiency, likely due to its crystallization behavior and limited protective interaction with the LNPs [36]. Also, gelatin-based (protein) excipient formulations form larger and less uniform particles with very low encapsulation efficiency, making them less ideal for the freeze–dried formulation.

### 4.7. Buffer-Dependent Modulation of pH, Ionic Strength, and LNP Integrity

Buffers also play a vital role during the freeze-drying of mRNA-LNPs vaccines [28,37]. They regulate the ionic environment as well as pH fluctuations in both the freezing and drying phases. This regulation helps maintain the native shape of the LNPs [15]. In this study, Tris and histidine consistently yielded smaller particle sizes and higher encapsulation efficiency during freeze-drying. Also, a recent study showed that 5 mM Tris maintains the physicochemical properties during the freeze-drying [10,16,38]. Tris and histidine buffers regulate the pH changes under acidic conditions in the presence of Mg^2+^ and Ca^2+^ [1,20,39,40]. Tris also protects the mRNA by scavenging hydroxyl radicals in the freeze-drying process [14]. On the other hand, PBS leads to a larger particle size and lower encapsulation efficiency. The negative effects of PBS buffer align with prior reports highlighting that the high ionic strength of PBS destabilizes the lipid layers [19]. PBS also undergoes substantial pH changes in the freeze-drying process when the temperature shifts from ambient chamber temperature to ultra-low temperature. Additionally, the histidine tested here for stability studies is one of its kind. Although it did not perform well with trehalose, the use of histidine with sucrose is an addition to the existing library of excipients.

### 4.8. Glass Transition and Vitrification Effects in Stabilizing mRNA-LNPs

This superior stability can be attributed to several well-documented mechanisms in the literature. Both sucrose and trehalose provide cryo- and lyoprotectant effects via a vitrification mechanism, forming an amorphous glassy matrix. The formation of an anhydrous glassy matrix by cryoprotectants like sucrose or trehalose immobilizes both mRNA and lipid components. This reduction in molecular mobility limits hydrolytic and oxidative reactions. Also, this rigid, vitrified structure subdues nanoparticle aggregation and fusion during storage [17,21,35]. Sucrose and trehalose interact with polar head groups on lipid bilayers and with the phosphate backbone of the RNA. This replaces water molecules through hydrogen bonding during freeze–drying stages. This hydrogen-bond network inhibits direct contact between lipids and ice crystals, reducing mechanical stress and sustaining the native arrangement of LNPs [17,21]. Additionally, these sugars form thin, amorphous coatings that protect encapsulated mRNA from exposure to environmental RNases [3,17,35,41]. Although this study did not analyze Karl Fischer titration to quantify residual moisture, differential scanning calorimetry (DSC) to determine glass transition temperature and molecular mobility, or X-ray diffraction (XRD) to assess cryoprotectant crystallization, future studies will incorporate these analytical methods to directly validate the proposed protective glassy matrix and degradation hypotheses.

### 4.9. Long-Term Stability of Freeze–Dried mRNA-LNPs at Subzero and Refrigerated Temperatures

In the long-term stability study, all freeze–dried formulations stored at −80 °C showed physicochemical stability of the mRNA-LNPs. This confirms that ultra-low temperature storage preserves the mRNA-LNPs’ integrity [29]. Storage at −80 °C is regarded as the gold standard for the long-term preservation of the structural integrity and function of mRNA-LNPs [3,15,42,43]. Also, freeze–dried formulations stored at −20 °C showed promising stability in terms of physicochemical properties and *in vitro* assays. These results correspond to similar mRNA-LNPs formulations, where standard freezer conditions enable acceptable shelf-life when combined with optimal excipients [44]. At such low temperatures, all chemical degradation processes, such as hydrolysis, oxidation, or enzymatic cleavage, are near zero, explaining the stability properties. As a result, mRNA remains highly intact, and the lipid nanoparticle structure is preserved, with minimal aggregation or leakage [14].

The freeze–dried mRNA-LNPs formulations also showed good stability at 4 °C in long-term storage. This reflects comparable data from Muramatsu et al. (2022), who showed that refrigerated lyophilized LNPs retain most physicochemical properties over months [29]. Also, the freeze–dried mRNA-LNPs formulations maintain the biological activity following four months at 4 °C.

### 4.10. Limitations of Ambient Temperature Storage (20 °C)

However, at 20 °C, all samples are found to be unstable with increased size, PDI, and marked loss in encapsulation. This instability was partially prevented by the freeze–dried formulations in the early stage (up to 3 months, in some formulations), but they could not fully prevent degradation or particle aggregation in the long run. These results align with reports from Meulewaeter et al. (2023) and Zhao et al. (2020), who found difficulty in maintaining the stability of mRNA-LNPs at ambient temperature [19,44].

Maintaining optimized freeze–dried mRNA-LNPs formulations at 20 °C is difficult because of accelerated degradation compared to storage at 4 °C. At 20 °C, residual moisture and increased molecular mobility within the lyophilized matrix led to enhanced rates of lipid oxidation and hydrolysis. This results in reduced encapsulation efficiency and aggregation or fusion of nanoparticles [3,7,11]. The protective glassy matrix formed by cryoprotectants becomes less effective at higher temperatures because of increased molecular diffusion and possible crystallization of excipients [20,21]. In this study, we clarify that these degradation mechanisms are inferred based on the observed stability trends in our study and supported by established literature, rather than directly measured. The absence of direct analytical measurements of residual moisture content and lipid chemical degradation is acknowledged as a limitation of the present work.

Therefore, although lyophilization improves stability, long-term storage at 20 °C remains challenging. This requires further formulation optimization or stabilization strategies to ensure longer shelf-life. Based on the tested formulations, the *in vitro* transfection study reveals that only F1 (20% sucrose + 5 mM Tris) retained the biological activity up to 4 months (Matrix, Figure 9). The probable mechanism is that the glassy matrix of sucrose maintained the functional integrity of mRNA-LNPs with a complementary effect by the tris, contributing to stability through pH buffering at this relatively high temperature.

## 5. Conclusions

In conclusion, this study confirms the critical role of cryoprotectants (sucrose and trehalose) and appropriate buffers (Tris and histidine) in stabilizing mRNA-LNPs during freeze-drying and prolonged storage at a wide range of temperatures. The novelty of this study is the identification of high-concentration (20%) sugars, specifically sucrose and trehalose, as effective stabilizers that better maintain mRNA-LNP size and functionality after freeze-drying. In addition, this work introduces histidine buffer as a novel buffer for freeze–dried formulations, showing enhanced performance alongside Tris. In addition, ultra-low temperature and refrigerated temperature maintain LNPs’ integrity and encapsulation effectively, and higher temperature stability remains subtle. These insights improve the understanding of excipient and buffer selection essential for advancing freeze-drying of the mRNA-LNPs formulation. Overall, these findings provide a strong foundation for rational formulation design, enabling the development of more robust, storage-stable mRNA-LNPs vaccine candidates suitable for global distribution.

## Figures and Tables

**Table 1:** Different formulations for the preliminary mRNA-LNPs freeze-drying study.

Buffer	Concentration	Excipients	Concentration	Formulations
Tris	5 mM	Sucrose	10%	1
		Sucrose	20%	2
		Trehalose	10%	3
		Trehalose	20%	4
		Mannitol	10%	5
		Mannitol	20%	6
		Gelatin	0.5%	7
		Gelatin	1%	8
Histidine	10 mM	Sucrose	10%	9
		Sucrose	20%	10
		Trehalose	10%	11
		Trehalose	20%	12
		Mannitol	10%	13
		Mannitol	20%	14
		Gelatin	0.5%	15
		Gelatin	1%	16
PBS	1 X	Sucrose	10%	17
		Sucrose	20%	18
		Trehalose	10%	19
		Trehalose	20%	20
		Mannitol	10%	21
		Mannitol	20%	22
		Gelatin	0.5%	23
		Gelatin	1%	24

**Table 2:** Different formulations for long-term stability.

F1	F2	F3	F4
Sucrose 20%	Sucrose 20%	Trehalose 20%	Trehalose 20%
Tris 5 mM	Histidine 10 mM	Tris 5 mM	Histidine 10 mM

**Table 3:** Freeze-drying parameters for mRNA-LNPs.

	Freezing	Primary Drying	Secondary Drying
Temperature	−50 °C	−30 °C	20 °C
Pressure	-	60 mTorr	30 mTorr

## Data Availability

The original contributions presented in the study are included in the article; further inquiries can be directed to the corresponding author.

## Supplementary Materials

The following supporting information can be downloaded at: https://www.mdpi.com/article/10.3390/vaccines14030242/s1, Supplementary Figure S1. Long-term mRNA integrity assessed by *in vitro* transfection of four mRNA-LNPs formulations stored at 4 °C and 20 °C; Supplementary Figure S2. Long-term stability of mRNA-LNPs formulations assessed by transfection efficiency.
