# Supplementary material for: Investigation on a Freeze-Drying Process for Long-Term Stability of mRNA-LNPs
Source: Vaccines (Basel). 2026 Mar 6;14(3):242. doi: 10.3390/vaccines14030242 (PMC13030275; doi:10.3390/vaccines14030242)
Supplement: Supplementary file 1 [file vaccines-14-00242-s001.zip › vaccines-4090103-supplementary.pdf]

vaccines

MDPI

Article

Investigation on a Freeze-Drying Process for Long Term Stability of mRNA-LNPs

MD Faizul Hussain Khan, Ayyappasamy Sudalaiyadum Perumal, and Amine A. Kamen.

Supplementary Material

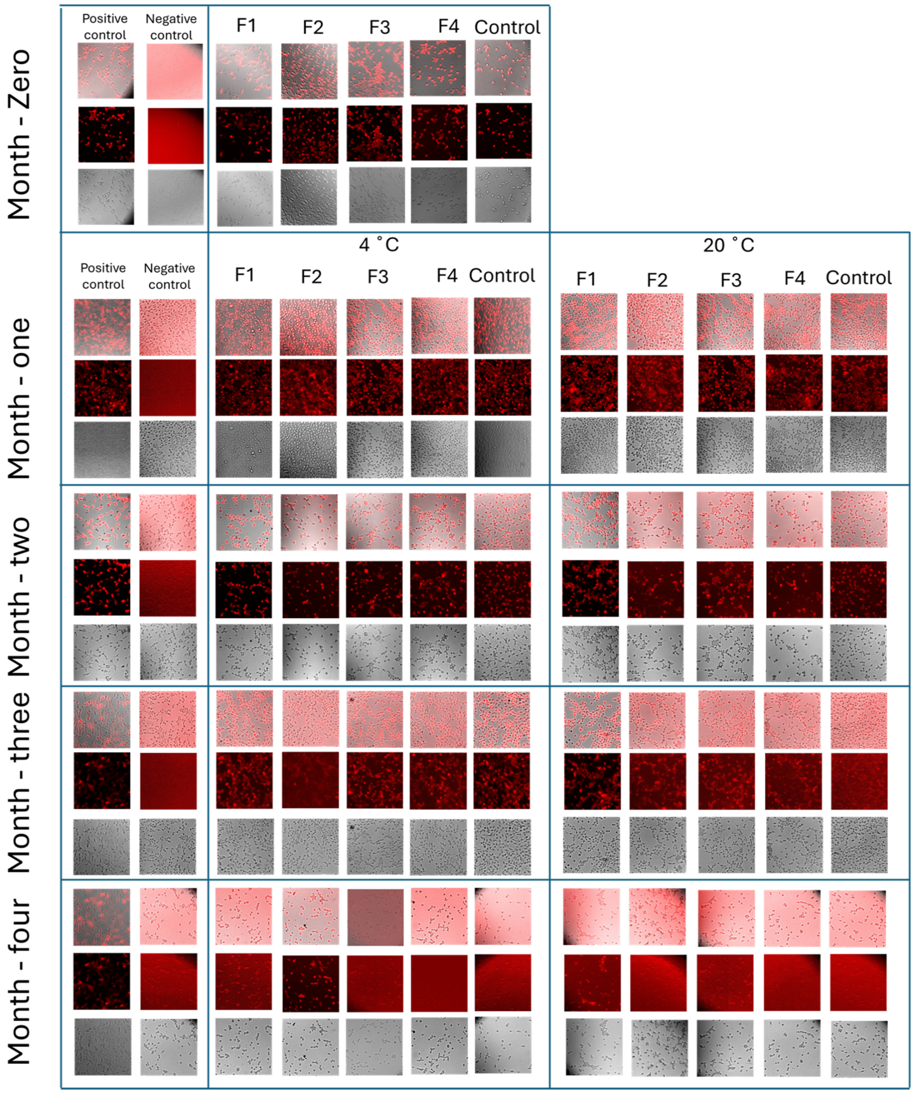

Commented [AS1]: Should we not include the paper title and author name without affiliation here ? This is standard practise for most journals and so just double checking. Double check if the format is okay here.

Commented [MK1R2]: I have added the title and author name. The editor will finalize the format.

vaccines

Supplementary Figure S1. Long-term mRNA integrity assessed by in vitro transfection of four mRNA-LNPs formulations stored at 4 °C and 20 °C. Transfection efficiency was quantified at one-month intervals using fluorescence microscopy–based image analysis. All experiments were performed in biological triplicates (*n* = 3); the images shown are representative of one replicate. For each time point, image panels include composite images (top row), fluorescence images (middle row), and bright-field images (bottom row). Month 0 corresponds to samples analyzed immediately after freeze-drying.

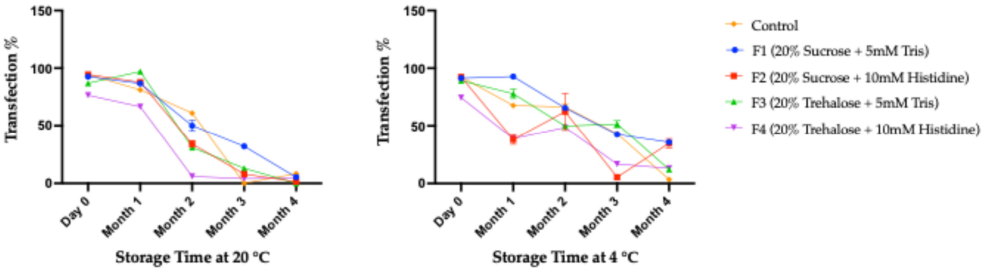

Supplementary Figure S2. Long-term stability of mRNA-LNPs formulations assessed by transfection efficiency. Four mRNA-LNPs formulations were stored at 4 °C and 20 °C, and in vitro transfection efficiency was monitored over time. Fluorescence microscopy–based image analysis was used to quantify transfection efficiency as the percentage of transfected cells at one-month intervals. Data are presented as mean ± standard deviation from biological replicates (*n* = 3). Transfection efficiencies of control samples are not shown; positive controls consistently exhibited ~100% transfection, while negative controls remained near 0%, as expected.
